# Predictors of poor health-related quality of life among hemodialysis patients with anemia in Jordan

**DOI:** 10.1186/s12955-021-01905-7

**Published:** 2021-12-24

**Authors:** Osama Y. Alshogran, Esraa A. Shatnawi, Shoroq M. Altawalbeh, Anan S. Jarab, Randa I. Farah

**Affiliations:** 1 Department of Clinical Pharmacy, Faculty of Pharmacy, Jordan University of Science and Technology, Irbid, 22110 Jordan; 2 Department of Internal Medicine, School of Medicine, The University of Jordan, Amman, Jordan

**Keywords:** Hemodialysis, Quality of life, EQ-5D-5L, Anemia, Predictors

## Abstract

**Objective:**

This study examined health-related quality of life (HRQoL) and factors associated with poor HRQoL among hemodialysis (HD) patients.

**Methods:**

A multicenter cross-sectional study was conducted on HD patients with anemia in Jordan (n = 168). Validated questionnaires were utilized to collect data on HRQoL using EQ-5D-5L, psychiatric symptoms using Hospital Anxiety and Depression Scale (HADS), and comorbidities score using the modified Charlson Comorbidity Index (mCCI). Multiple linear regression analysis was conducted to identify the variables which are independently associated with HRQoL among patients.

**Results:**

The mean (± SD) age of study participants was 52.2 (± 14.6) years. The mean utility value of EQ-5D-5L was 0.44 (± 0.42). Participants reported extreme problems mostly in pain/discomfort domain (19.6%). Increased age, increased mCCI and patient complains, more years under dialysis, decreased exercise, and low family income were significantly associated with poor HRQoL (*p* < 0.05).

**Conclusion:**

The study findings revealed poor HRQoL among HD patients with anemia. Various dimensions of health were negatively affected among HD patients. Development and implementation of appropriate approaches with adequate education and psychosocial support to HD patients by healthcare professionals targeting improved HRQoL and clinical outcomes would be necessary.

## Introduction

Chronic kidney disease (CKD) is a common global health problem. End-stage renal disease (ESRD) represents the ultimate progressive stage of CKD where patients are primarily dependent on hemodialysis (HD) as part of renal replacement therapy [1]. Patients with ESRD have multiple co-morbid conditions including diabetes and hypertension, and are more prone to renal-related complications such as imbalance in calcium and phosphate homeostasis, vitamin D deficiency, anemia, and secondary hyperparathyroidism [2–4], which together may significantly impact patients’ well-being and health outcomes. Additionally, maintenance dialysis by itself is an invasive procedure which subjects patients to additional pain and discomfort [5]. Anemia is prevalent in CKD, especially in patients receiving dialysis [6, 7]. Iron supplementations and erythropoiesis-stimulating agents (ESA) are the main treatment modalities for anemia in HD patients [8].

Health-related quality of life (HRQoL) is considered a multi-dimensional ultimate health outcome measure which should be frequently monitored in patients with ESRD [9]. Several studies documented poor HRQoL among ESRD patients [10–13], with lower HRQoL being reported among CKD patients with anemia [14]. An earlier study showed that HD patients with anemia who received high ESA dosing or intravenous iron therapy had slightly better HRQoL scores after 1–3 months follow-up period [15]. Poor HRQoL was linked to increased hospitalization and mortality among ESRD patients [9, 16]. Therefore, one of the primary goals of ESRD management is to implement interventions that improve HRQoL. Compromised HRQoL among ESRD patients could be associated with physical, social, psychological and adherence factors [17, 18].

The primary treatment modality for ESRD patients in Jordan is HD. Based on the Jordan Renal Registry data, the total number of ESRD patients in Jordan in 2019 was 6708. Of them, 6316 patients were treated in dialysis units. A total of 6165 (97.6%) were receiving HD, while only 151 patients (2.4%) were undergoing peritoneal dialysis. The majority of patients in Jordan were males (63%) with age of more than 40 years old (76%). To date, limited information exists about variables associated with poor HRQoL among HD patients with anemia, particularly in Jordan. Thus, this study was conducted to evaluate HRQoL among a representative group of HD patients with anemia in Jordan, and the independent associations of multiple factors with poor HRQoL.

## Methods

### Study design and setting

This cross-sectional study was conducted on HD patients at different dialysis units in Jordan from January through October 2018. Patients who visit HD clinics at King Abdullah University Hospital (KAUH), Jordan University Hospital (JUH), Princess Basma Hospital, and Al-Ramtha Governmental Hospital were approached during their scheduled dialysis sessions. The inclusion criteria included HD patients older than 18 years who have been under dialysis for at least three months and able to communicate. After screening for eligibility, patients were informed about study objectives and invited to participate by a trained clinical pharmacist. Patients were assured about the right to withdraw and that the collected information will be kept confidential. All participants signed a written informed consent. Patient’s interview was conducted after 30–60 min of the start of dialysis, with an average interview time of 15–20 min.

### Sociodemographics and clinical data

Socio-demographics including age, gender, weight and height, material status, educational level, employment, family status, family income per month, and smoking status were collected. Medical files were also used to collect information on cause of ESRD, dialysis access, time since first dialysis, dialysis sessions per week, length of dialysis session, current medications, and patient major complains. Biochemical data of hemoglobin (Hgb), ferritin, creatinine, urea, calcium, potassium, parathyroid hormone, albumin, were also obtained from medical reports.

### Study instruments

The primary outcome that was measured in the present study was subject’s HRQoL and was assessed using the generic EQ-5D-5L instrument developed by Euro QOL Group. Participants were asked to complete an Arabic version of the EQ-5D-5L questionnaire which was obtained from the developer with permission (ID: 21432). The first part of the instrument is a descriptive system of patient’s HRQoL in terms of five dimensions of health including mobility, self-care, usual activities, pain/discomfort, and anxiety/depression. The answers for each dimension were rated on a five–level scale of no problems, slight problems, moderate problems, severe problems and unable to/extreme problems. The collected responses were then scored to calculate the index value using the value sets (weights) from United Kingdom general population scoring algorithm (i.e. EQ-5D-5L Crosswalk Index Value Calculator) [19]. The index value for each individual could range from -0.594 to 1. Higher index values indicate better quality of life and vice versa.

The second part of the instrument is the EQ Visual Analogue Scale (EQ_VAS) which was used to evaluate patients’ self-rated health states on a rating scale ranging from 0 to 100, with higher scores indicating better quality of life level [20].

The EQ-5D is a reliable scale that has been validated in Arabic language [21, 22]. The instrument has been widely used among different disease conditions in Jordan [23, 24] and was implemented previously among HD patients [14, 25].

A previously validated and highly-implemented Arabic version of the hospital anxiety and depression scale (HADS) was utilized to explore psychiatric symptoms among study participants [26, 27]. This instrument contains seven items for each subscale of anxiety and depression, with scores ranging between 0 and 21 for each subscale. High scores indicate more psychiatric distress. The modified Charlson comorbidity index (mCCI) in ESRD which includes 18 comorbidities was used to calculate patients' comorbidity score as previously described [28]. Each disease condition was assigned a specific weight that ranges between 1 and 10. For example, dementia was given a weight of 1 while the presence of metastatic disease was given a weight of 10 [28]. The score for each patient was generated according to his/her comorbidities, then the mean score of the study sample was calculated. Higher scores indicate higher comorbidity burden. Additionally, information about major patients complains that might be related to disease or medications as well as knowledge about indication of prescribed medications were collected from participants.

The questionnaire was initially developed by the research investigators based on previous literature as described earlier. A pilot study was then conducted among a group of HD patients (n = 10) to ensure that the questionnaire items are understandable and clear to study subjects. The developed survey was generally clear to the pilot subjects with few recommendations to rephrase some of questions and make the survey a little shorter. The questionnaire items were then updated based on patients’ suggestions. The questionnaire was retested among a couple of patients to ensure clarity. Data of the pilot study were not included in the final analysis.

### Statistical analysis

Patient’ demographics and clinical characteristics were presented using simple descriptive statistics. Categorical data were listed as counts and percentages while continuous data were reported as arithmetic means with standard deviation. Multivariable linear regression was conducted to identify variables associated with poor HRQoL. Variables (sociodemographic, clinical, medical, labs, etc.) included in regression models were selected using backward stepwise process with a *P* < 0.2 to stay. Data analyses were conducted using STATA version 14 (StataCorp. 2015. Stata. Statistical Software: Release 14. College Station, TX: StataCorp LP.)

## Results

### Patients socio-demographics and clinical characteristics

A total of 245 patients were approached and invited to participate. Eleven subjects were excluded as they did not match the criteria and 66 patients refused to participate. Accordingly, a total of 168 patients were interviewed by the research pharmacist and entered to the final analysis, giving a response rate of 71.8%. More than half of the patients (59.5%) were male. Participants mean (± SD) age was 52.2 (± 14.6) years, and ranged from 18 to 85 years. A quarter of the patients were obese (25%), half were current smoker or ex-smoker (50.6%), and the majority were married (66.7%). The majority of participants were unemployed (85.7%) and had up to school education level (72.6%). Other socio-demographic characteristics are listed in Table 1.

**Table 1:** Socio-demographic features of the studied population

Variable	Number (%) N = 168
*Age (years)*	
18–40	35 (20.8)
40–64	97 (57.7)
> 65	36 (21.4)
*Gender*	
Male	100 (59.5)
Female	68 (40.5)
*BMI (kg/m*^*2*^*)*	
Underweight < 18.5	9 (5.4)
Normal range 18.5–24.9	73 (43.5)
Overweight 25–29.9	44 (26.2)
Obese > 30	42 (25.0)
*Marital status*	
Single	34 (20.2)
Married	112 (66.7)
Divorced or widow	22 (13.1)
*Education level*	
Illiterate	10 (5.9)
Junior school	50 (29.8)
High school	62 (36.9)
College or over	46 (27.4)
*Employment status*	
Employed	24 (14.3)
Unemployed	144 (85.7)
*Family status*	
Live alone	8 (4.8)
Live with partner	15 (8.9)
Live with family	137 (81.6)
Others	8 (4.8)
*Family income (month)*	
Low (less than 250 JD)	52 (30.9)
Moderate (250–500 JD)	83 (49.4)
High (more than 500 JD)	33 (19.7)
*Smoking status*	
Smoker	50 (29.8)
Nonsmoker	83 (49.4)
Ex-smoker	35 (20.8)
*Co-morbidities*	
Hypertension	113 (67.3)
Diabetes mellitus	68 (40.9)

BMI, Body Mass Index

Regarding subjects’ clinical and medical characteristics (Table 2), self-reported diabetes was the main etiology for ESRD in the study (32.9%) followed by hypertension (30.4%). The majority of the patients (89%) were prescribed iron therapy including oral formulation such as ferrous gluconate, ferrous sulphate, and iron complex or intravenous products as iron sucrose and iron dextran. Most of the patients (> 75%%) were also prescribed erythropoietin, vitamin D, and calcium carbonate. Only about half of them were aware about the indication for use of such medications (47.4% for iron and 50.2% for erythropoietin). The majority of the patients (90.5%) received dialysis three times a week, of which (69.6%) were performed using fistula as a vascular access. Furthermore, most of patients (69.6%) are on dialysis for more than two years. The mean (± SD) mCCI score for participants was 4.22 (± 3.62) which ranged between zero and 18. The major comorbid conditions observed among our sample were diabetes (40.5%) and peripheral vascular disease (32.1%). The mean (± SD) Hgb level was 10.27 (± 1.59) g/dL, and the mean iron level was 9.43 (± 11.46) μmol/L. Other patient's laboratory data which were collected at the time of interview are shown in the Table 2.

**Table 2:** Patients clinical, medical and laboratory information

Variable	N (%)
Cause of ESRD	
HTN	38 (22.8)
DM	43 (25.8)
HTN and DM	12 (7.2)
Glomerulonephritis	5 (2.9)
Others	2 (1.2)
Drugs	1 (0.6)
Medical error	66 (39.3)
Unknown	69 (41.7)
Number of prescribed medications	
< 5	19 (11.6)
5–10	90 (54.9)
> 10	55 (33.5)
Vascular access for HD	
Catheter	39 (23.2)
Fistula	117 (69.6)
Graft	12 (7.2)
Dialysis sessions per week	
2 Times weekly	16 (9.5)
3 Times weekly	152 (90.5)
Length of dialysis session in hours	
3 h or less	32 (19.1)
3.5 h	57 (33.9)
4 h or more	79 (47.0)
Years under dialysis	
< 2 years	51 (30.4)
2–5 years	52 (30.9)
> 5 years	65 (38.7)
Patients on iron products	149 (88.7)
Patients on erythropoietin	159 (94.6)
Psychiatric symptoms^a^	
HADS-A	5.2 (± 4.5)
HADS-D	6.4 (± 4.9)
HADS-T	11.6 (± 8.9)
Patients had practiced exercise last week	37 (22.16)
Duration of exercise per week in minutes^a^	148.11 (± 126.3)
Number of emergency visits last year^a^	2.94 (± 4.18)
Number of hospital admissions last year^a^	1.66 (± 2.19)
Serum creatinine^a^ (normal range 0.7–1.3 mg/dL)	9.22 (± 2.69)
Serum urea^#^ (normal range: 2.5–7.1 mmol/L)	21.36 (± 6.76)
Calcium level^a^ (normal range 2.2–2.6 mmol/L)	2.18 (± 0.23)
Potassium level^a^ (normal range 3.5–5.0 mmol/L)	5.29 (± 0.83)
Parathyroid hormone^a^ (normal range 10–65 ng/L)	633.7 (± 626.8)
Iron^a^ (normal range 11–29 μmol/L)	9.43 (± 11.46)
Hemoglobin^a^ (normal range male 14–17, female 12–16 g/dL)	10.27 (± 1.59)
Total iron binding capacity^a^ (normal range 250–460 μg/dL)	233.97 (± 45.47)
Serum ferritin^a^ (normal range 15–200 ng/mL)	458.58 (± 640.36)

HTN, hypertension; DM, diabetes mellitus; HADS-A, hospital anxiety and depression scale-anxiety; HADS-D, hospital anxiety and depression scale-depression; HADS-T, hospital anxiety and depression scale-total

^a^Data are presented as mean (± SD)

About a quarter of patients (22.6%) had severe gastrointestinal symptoms during the last week of interview. One-third (32.7%) of patients reported severe joint and muscle pain and 32.7% of patients had severe insomnia. Overall, less than half of participants were aware about the indication of prescribed medications.

### Quality of life and health status

**Fig. 1:**
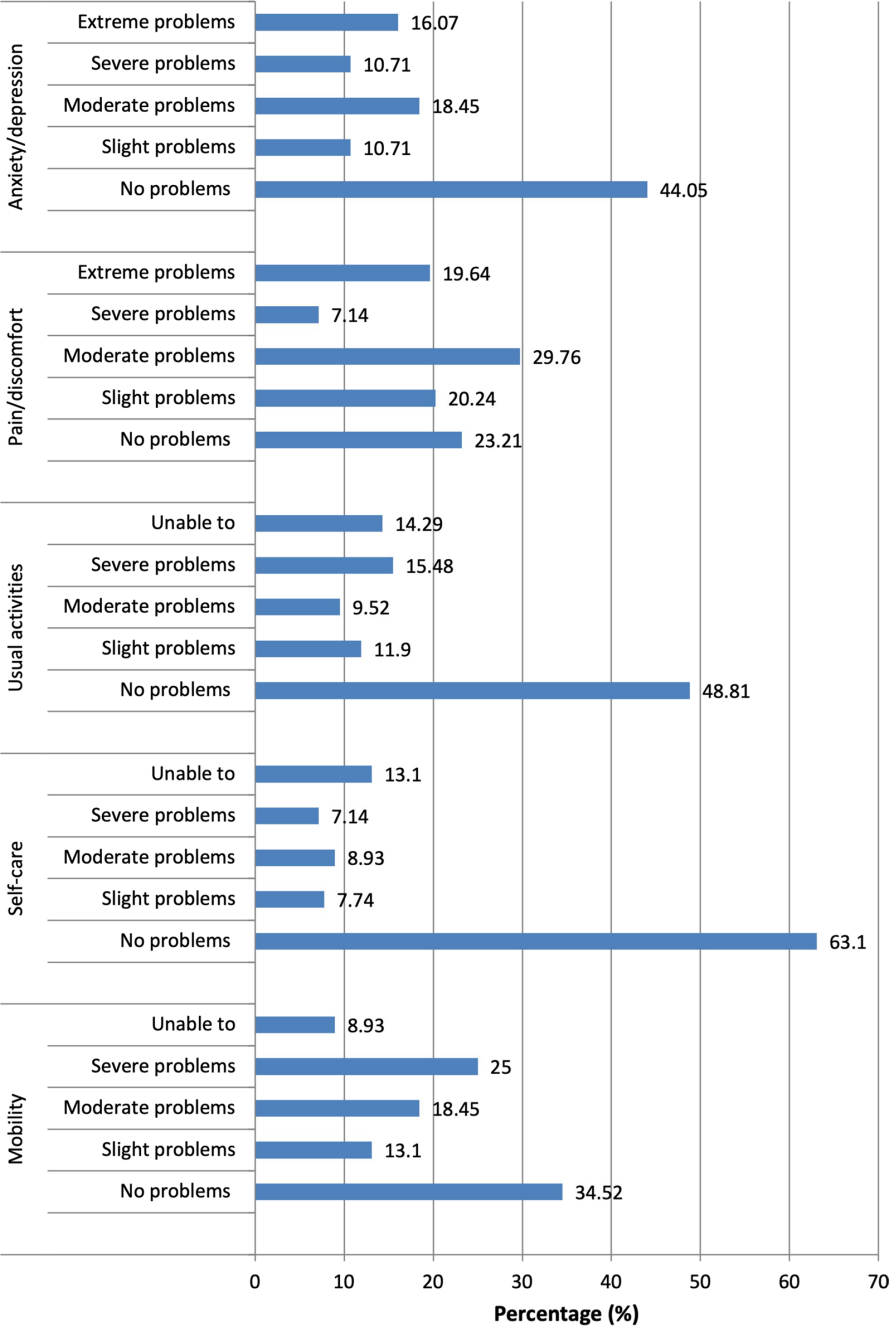
The distribution of health-related quality of life measures using (EQ-5D-5L)

The EQ-5D-5L results are shown in Fig. 1. The dimension that showed the highest response rate of “no problems” was “self-care” (63.1%). While, the dimension with the lowest response rate for “no problems” was “pain/discomfort” (23.2%). The mean utility or index score (± SD) was 0.44(± 0.42), ranging from -0.594 to 1.0. The number (%) of patients who answered “extreme problem or unable to” for the five items of EQ-5D-5L was as follows: anxiety/depression 27 (16.07%), pain/discomfort 33 (19.64%), usual activities 24 (14.29%), self-care 22 (13.10%) and mobility 15 (8.93%). The mean EQ-VAS (± SD) value was 62.05 ± 22.61.

The results of multivariate regression analysis revealed that increased age, longer years under dialysis, increased comorbidities (mCCI), and patients general complains were significantly and negatively associated with HRQoL, while increased income and doing exercise were positively associated with HRQoL (Table 3). For example, one unit increase in the mCCI score was significantly associated with a decrease in HRQoL index score by − 0.026.

**Table 3:** Predictors of quality of life among HD patients

Variable	Beta	(CI) 95%	*P* value*
Age	− 0.0779	(− 0.1549 to − 0.0085)	0.048
Income per month			
< 250 JD	Ref	Ref	Ref
250–500 JD	0.0544	(− 0.04115 to 0.1499)	0.263
> 500 JD	0.1871	(− 0.0411 to 0.1499)	0.003
Exercise	0.1571	(0.0538–0.2605)	0.003
Years under dialysis			
< 2	Ref	Ref	Ref
2–5	− 0.1017	(− 0.2071 to 0.0037)	0.058
> 5	− 0.1982	(− 0.3043 to − 0.920)	< 0.001
Comorbidity index	− 0.0259	(− 0.0407 to − 0.0111)	0.001
Average patients complains	− 0.3225	(− 0.4060 to 0.2389	< 0.001

Variable included in the model were selected using backward stepwise process with *P* < 0.2 to stay

*Statistical significance of multivariate regression analysis was set at a 2-sided *P* < 0.05

## Discussion

The current study investigated HRQoL and its associated variables in patients with HD in Jordan. Results clearly suggest compromised HRQoL among study participants with the worst health status for pain/discomfort and mobility dimensions. Longer years under dialysis, increased age, increased number of comorbid conditions and patients’ major complaints were associated with poor HRQoL, while higher income and conducting exercise were associated with better HRQoL.

Although HRQoL among HD patients has been investigated at earlier time in Jordan [18, 29], the current study is the first one to use the EQ-5D-5L to explore HRQoL among HD patients in Jordan. The latter instrument is simple, easy to be completed, well recognized, requires shorter time to be finished, and has enhanced sensitiviy as compared with other available instruments for measuring HRQoL among patient population [13, 30]. We found that the mean index (± SD) value among HD patients was 0.44 (± 0.42), while findings from other studies that used the same instrument in Palestine, Japan and Korea found utility values of 0.37 (± 0.440) [13], 0.809 (± 0.184) [31] and 0.704 (± 0.199) [32] respectively. This variation in HRQoL could be related partially to differences in socio-demographic and clinical characteristics including age, HD duration, and the presence of comorbid conditions in the recruited sample.

An earlier study conducted in Jordan reported a moderately decreased level of HRQoL among ESRD patients using Quality of Life Index-Dialysis Version III (QLI-DVIII) [18]. The latter study also revealed adherence to treatment, depression, and perceived seriousness of illness as significant predictors of HRQoL [18]. Another study used the Quality of Life Index showed that HD patients in Jordan had moderate to high life satisfaction [29]. Shdaifat et al. used the RAND 36-Item Health Survey in Jordan which showed that patients were found to have lower HRQoL when compared to their caregivers and to the general population [33].

Consistent with earlier research findings [13, 32], results in the present study showed that increased age was significantly associated with lower HRQoL. For example, Zyoud et al. [13] revealed that younger patients (< 30 years) were found to have significantly better HRQoL than older one using the EQ-5D-5L instrument. This could be attributed partially to the lower physical activity and to the increased number of comorbid conditions in old patients [34].

In agreement with the findings of previous studies [12, 13, 35, 36], the current study revealed a negative association between comorbidity index and HRQoL. For instance, a study conducted among Singaporean patients with renal failure documented that low Charlson comorbidity index was significantly linked with better HRQoL [35]. This association could be explained by the burden of increased number of comorbid conditions as well as the need for a higher number of medications and their associated negative consequences on health.

In contrast to the present research findings, Yang et al. [35] reported similar or better mental health status among patients with longer dialysis vintage versus those with shorter dialysis vintage. Our finding of the assocaition between decreased quality of life with longer dialysis years, could be attributed to increased economic burden and dialysis complications with longer years under dialysis. The observation that exercise acts as a positive predictor of HRQoL is in agreement with Tsai et al. finding [37]. Physical activity levels were shown to be lower in dialysis patients compared to healthy sedentary controls [38]. Furthermore, exercise may improve the physical function as one of the dimensions of quality of life.

While Khatib et al. reported no association between household income and HRQoL [25], other studies demonestrated an improved HRQoL with higher income in terms of physical functioning, pain and social aspects (11, 39), which is in agreement with the current study finding. Possibly, patients who have higher income tend to have more leisure time and better social life than those who have low income.

Overall, the study findings provide important implications in identifying factors that could compromise HRQoL of HD patients. Targeting modifiable factors should be an essential component in any future appropriate interventions aiming at enhanced HRQoL of patients undergoing HD. Learning about such factors is important to improve patient care.

Our results provide insights into implementing procedures to improve health outcomes in dialysis patients. The current study highlights the needs of healthcare providers to develop and implement effective strategies for HD patients to help them improving their quality of life. For instance, early and continous assessment of QOL is critical. Healthcare professionals may establish teaching programs that provide patients with knowledge about the seriousness of the disease and the positive impacts of improved adherence. Also, development of support groups including patients and a multidisciplanary team of nephrologists, nurses, pharmacists and social workers would be necessary to improve treatment outcomes.

This study has many strengths. It is the first to assess HRQoL among HD patients with anemia in Jordan. The study utilized face-to-face interviews conducted by one clinical pharmacist which ensure consistently in data collection. Also, the current study explored various factors that may impact HRQoL such as socio-demographics, medical, laboratory, and psychiatric measures. However, study limitations should be noted. The cross-sectional design is descriptive and not intended to identify a cause-effect relationship between variables and outcomes. The recruited sample was only from four centers in Jordan which affects the generalizability of the study findings. Also, psychiatric information using HADS and some of iron indices data were not available for all subjects. Nevertheless, the current investigation has a unique conribution to the litertaure as it provides necessary baseline information about variables associated with poor HRQoL among anemic HD patients.

## Conclusions

The study elucidated poor HRQoL among HD patients with anemia. Various range of dimensions of life were negatively affected among HD patients, highlighting the need of the healthcare providers to develop and implement potential strategies that would improve QOL of this patient population. Future research should be directed towards well-designed prospective interventional studies that explore the efficacy of such approaches in improving health outcomes among HD patients.

## Data Availability

The data that support the findings of this study are available on request from the corresponding author. The data are not publicly available due to privacy or ethical restrictions.
